# Bioprocess development of antibody-drug conjugate production for cancer treatment

**DOI:** 10.1371/journal.pone.0206246

**Published:** 2018-10-23

**Authors:** Jianfa Ou, Yingnan Si, KahYong Goh, Norio Yasui, Yichen Guo, Jiajia Song, Lizhong Wang, Renata Jaskula-Sztul, Jinda Fan, Lufang Zhou, Runhua Liu, Xiaoguang Liu

**Affiliations:** 1 Department of Biomedical Engineering, University of Alabama at Birmingham, Birmingham, Alabama, United States of America; 2 Department of Radiology, University of Alabama at Birmingham, Birmingham, Alabama, United States of America; 3 School of Medicine, University of Alabama at Birmingham, Birmingham, Alabama, United States of America; 4 Department of Surgery, University of Alabama at Birmingham, Birmingham, Alabama, United States of America; University of South Alabama Mitchell Cancer Institute, UNITED STATES

## Abstract

Antibody-drug conjugate (ADC) is a class of targeted cancer therapies that combine the advantages of monoclonal antibody (mAb)’s specific targeting and chemotherapy’s potent cytotoxicity. The therapeutic effect of ADC is significantly affected by its bioproduction process. This study aims to develop an effective ADC production process using anti-HER2 mAb-drug as a model therapeutic. First, a high titer (>2 g/L) of mAb was produced by Chinese hamster ovary cells from fed-batch cell culture. Both live-cell confocal microscopy imaging and flow cytometry analysis demonstrated that the produced mAb and ADC had strong and specific binding to HER2^+^ cell line BT474. Second, various conjugation conditions of mAb and drug, including linker selection, ratio of drug and mAb, and conjugation approaches, were investigated to improve the production yield and product quality. Finally, the ADC structure and biological quality were evaluated by SDS-PAGE and anti-breast cancer toxicity study, respectively. The ADC with integral molecular structure and high cytotoxicity (IC_50_ of 1.95 nM) was produced using the optimized production process. The robust bioproduction process could guide the development of ADC-based biopharmaceuticals.

## Introduction

As an effective targeted therapy, antibody-drug conjugate (ADC) has been developed to treat solid tumors while minimizing the side effects on normal cells [1–3]. It drew great attention after the first ADC, gemtuzumab ozogamicin (Mylotarg) for acute myelocytic leukemia treatment, was approved by the FDA in 2000 [4]. The high clinical need led to two recently approved ADCs, i.e. the CD30-targeting Brentuximab vedotin (Adcetris) to treat relapsed Hodgkin lymphoma and systemic anaplastic large cell lymphoma and HER2-targeting Trastuzumab emtansine (Kadcyla) to treat relapsed or chemotherapy refractory HER2^+^ breast cancer [5, 6]. Nowadays there are nearly 60 ADCs in clinical trials and this number continues to grow [7].

ADC is typically composed of monoclonal antibody (mAb), spacer or linker, and cytotoxic reagent or payload. The mAb enables ADC to circulate in the bloodstream until it binds to the tumor specific surface antigen. After binding, ADC is internalized via the receptor-mediated endocytosis, forms late endosome, undergoes lysosomal degradation, releases the toxic drug into the cytoplasm, and eventually leads to cancer cell death [8–10]. The challenges in ADC construction include: 1) high-quality mAb that specifically targets and delivers drugs to cancer cells, 2) suitable linker which is stable in circulation but quickly releases the payload after endocytosis, and 3) efficient and robust conjugation process to achieve high biological activity, high stability and reduced heterogeneity [11]. Two conjugation approaches, lysine- and cysteine-based, were developed to produce ADC. In lysine-based conjugation, the potent small molecule can directly react with antibody through the modified lysine while it needs accurate process control to reduce batch-to-batch variation and product heterogeneity [12, 13]. In cysteine-based conjugation, the cytotoxic drug can conjugate with the thiols generated from disulfide bond reduction, but it is important to use site-specific conjugation or novel linker to achieve high stability and structural integrity of ADC [14, 15]. In addition to conjugation process, the high-quality mAb production and potent free drug selection are also very important for ADC production.

The objective of this study was to develop an effective and robust bioproduction process of ADC. Several key parameters, i.e. mAb production, linker selection, conjugation conditions, and end product purification, were investigated. The HER2-targting ADC was used as a model biopharmaceutical. Both the molecular integrity and the anti-breast cancer toxicity of constructed ADCs were evaluated. The data collected in this study could benefit the ADC-based anti-cancer therapy development.

## Materials and methods

### Cell lines and cell culture

The seed culture of our in-house CHO DG44/anti-HER2 mAb was maintained in Dynamis medium, supplemented with 8 mM L-glutamine, 500 nM methotrexate and anti-clumping agent (0.3% v/v) in 125-mL shaker flask at 37 ^o^C, 5% CO_2_ and 130 rpm in a humidified incubator (Caron, Marietta, OH). Methotrexate was removed one passage before the mAb production in bioreactor. The HER2^+^ human breast cancer cell line BT474 (ATCC, Manassas, VA) was cultivated in DMEM/F12 medium supplemented with 10% fetal bovine serum (FBS) and 4 mM L-glutamine in T25 flask. The control cell line MDA-MB-231 (ATCC) was grown in DMEM containing 10% FBS and 4 mM L-glutamine in T25 flask. All basal media, supplements and reagents used in this study were purchased from Thermo Fisher Scientific (Waltham, MA) unless otherwise specified.

### Optimization of anti-HER2 mAb production

#### Bioproduction optimization

The established procedure of fed-batch cell culture in 2-L stirred-tank bioreactor for mAb production was described in our previous publication [16]. The mAb production cultures were seeded with viable cell density (VCD) of 0.3–0.5×10^6^ cells/mL in Dynamis medium supplemented with 6 g/L glucose and 8 mM glutamine. The nutrient EfficientFeeding C+ was fed to the cell culture broth on Day 3, 5, 7 and 9 during mAb production. The bioreactor production process parameters were controlled at 37°C, pH 7.0, DO 70% and agitation 70 rpm. The bioreactor was sampled daily to monitor cell growth, glucose, glutamine and anti-HER2 mAb titer. The VCD and viability were measured by cell counter (Thermo Fisher Scientific). Glucose concentration was measured by HemoCue Glucose 201 DM System, glutamine concentration was analyzed using YSI (YSI, Yellow Springs, OH), and mAb was titrated using a Bio-Rad NGC system. The glucose concentration was maintained at 2–6 g/L and glutamine concentration was maintained at 2–8 mM through feeding concentrated solution of glucose and glutamine, respectively. The mAb production was stopped when viability reached <50%, and antibody was harvested by centrifugation and filtration for purification. Similar fed-batch culture was performed in shaker flask at 37 ^o^C, 5% CO_2_ and 130 rpm in a humidified incubator without pH and DO control.

#### Purification and evaluation

Small-scale anti-HER2 mAb was purified using NAb Protein A Plus Spin Kit. Large-scale mAb purification using Bio-Rad NGC system (Bio-Rad, Hercules, CA) equipped with a UNOsphere SUPrA column was conducted according to the manufacturer’s protocols, including column equilibration, sample loading, column washing, and antibody elution. The equilibration buffer was comprised of 0.02 M sodium phosphate and 0.02 M sodium citrate at pH 7.5. Elution buffer contained 0.02 M sodium citrate and 0.1 M sodium chloride at pH 3.0. The pH of eluted mAb was neutralized to 7.0 with 1 M Tris solution. The mAb purity was examined by SDS-PAGE under natural condition using NuPAGE 4–12% Bis-Tris Protein Gels (1.0 mm, 10-well) and a PowerPac Basic Power Supply (Bio-Rad). The surface binding of our anti-HER2 mAb was analyzed using BD LSRII flow cytometer (BD Biosciences, San Jose, CA) after incubating cells with 1 μg of Alexa Fluor 488-labeled mAb/million cells on ice for 30 min.

### Bioprocess development of ADC construction

**Fig 1:**
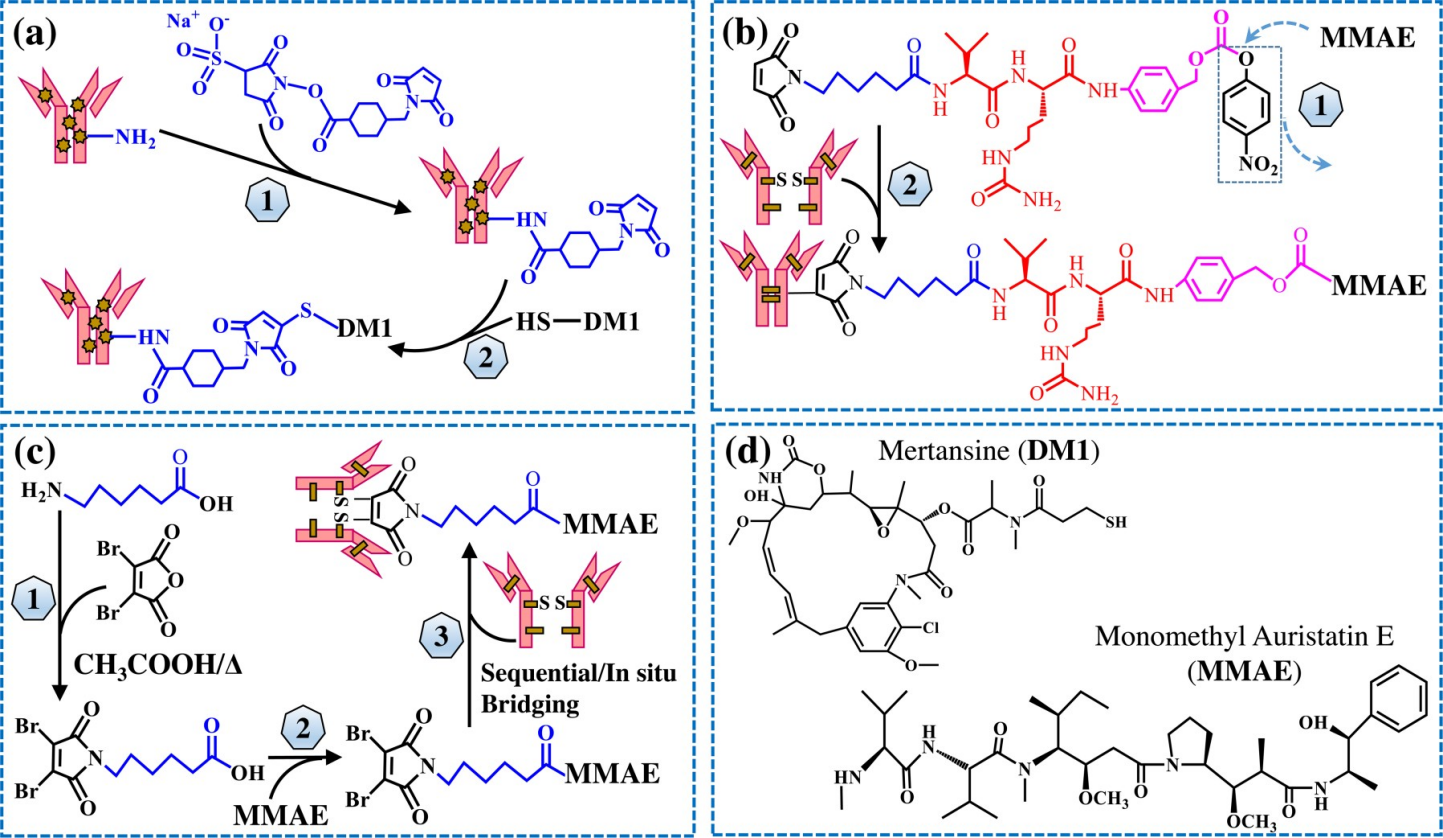
The diagram of ADC construction. (**a**) Lysine-based conjugation of mAb-DM1: 1) mAb modification by cross linker Sulfo-SMCC, and 2) conjugation of DM1 with the purified mAb-SMCC. (**b**) Cysteine-based conjugation of mAb-MMAE using traditional linker: 1) synthesis of Mc-Val-Cit-PABC-PNP linker-MMAE payload, and 2) conjugation of payload with the DTT/TCEP reduced mAb. (**c**) Cysteine-based conjugation of mAb-MMAE using rebridging linker: 1) synthesis of rebridging linker, 2) synthesis of rebridging linker-MMAE payload, and 3) conjugation of payload with the DTT/TCEP reduced mAb. (**d**) Structures of free drugs MMAE and DM1.

The diagram of ADC construction is shown in Fig 1, including both lysine-based conjugation and cysteine-based conjugation.

#### Synthesis of rebridging linker

The rebridging linker was synthesized following the published protocol [17] with minor modification. Briefly, 3.91 mmol 6-aminohexanoic acid was mixed with 3.91 mmol 3,4-dibromofuran-2,5-dione in 20 mL of acetic acid. After stirring for 10 min at room temperature, the solution was heated at 100°C for 18 h. The solvent was removed by vacuum and the rebridging linker was purified with silica gel with eluent solution of dichloromethane/ethyl acetate 0–40%.

#### Synthesis of linker-MMAE payload

The peptide-based traditional Maleimidocaproyl(Mc)-Val-Cit-PABC-PNP linker or the rebridging linker were reacted with monomethyl auristatin E (MMAE). In the construction of traditional linker-MMAE payload, 18.20 μmol potent molecule MMAE, 16.38 μmol Mc-Val-Cit-PABC-PNP, and 3.64 μmol hydroxybenzotriazole were dissolved and mixed in 500 μL dimethylformamide. Then 18.20 μmol pyridine was added to the mixture after 2 min, and 20 μmol trifluoroacetic acid (TFA) was added after 24 h. In the construction of rebridging linker-MMAE payload, 13.55 μmol N,N'-diisopropylcarbodiimide, 13.55 μmol N,N-diisopropylethylamine, and 33.85 μmol synthesized rebridging linker were mixed in 0.25 mL dichloromethane, followed by frequent mixing for 1 h at room temperature. Then 13.55 μmol MMAE was added and frequently mixed for additional 16 h. After linker-MMAE conjugates were synthesized, the solvents were removed by vacuum pump and the conjugates were purified by a Waters HPLC system equipped with 600 Controller/Pump and 996 PDA detector (Waters, Milford, MA). A reversed-phase C_18_ column with 5 μm C18(2) 100 Å and 250 x 10 mm (Phenomenex Luna; Torrance, CA) was used with gradient elution buffer of Phase A (water+0.1% TFA) and Phase B (acetonitrile). The purified products were confirmed by Agilent 6500 Series Accurate-Mass Q-TOF LC/MS (Agilent Technologies, Santa Clara, CA).

#### ADC production

The anti-HER2 mAb produced in this study was used to generate all conjugates. The lysine-based ADC was produced following a previously developed method with modification [18]. Briefly, the crosslinker Sulfo-SMCC was mixed with 5 mg/mL mAb in PBS and incubated for 30 min at room temperature. The excessive crosslinker was removed by repeated buffer exchange using Pierce Protein Concentrator. Then with cytotoxic mertansine (DM1) reacted with the SMCC-modified mAb at different molar ratios (4:1, 8:1 and 16:1) for 30 min. The final product was purified by PD MidiTrap G-25 (GE Healthcare, Little Chalfont, United Kingdom) gel filtration.

The cysteine-based ADC was constructed using two conjugation approaches, i.e. sequential conjugation and *in situ* conjugation [19, 20]. In sequential conjugation, 5 mg/mL mAb solved in 50 mM borate buffer at pH 8.0 was reduced with 1 mM dithiothreitol (DTT) at 37°C for 1 h, followed by repeated buffer exchange in Pierce dialysis column using PBS buffer containing 1 mM pentetic acid. Then the traditional linker-MMAE and rebridging linker-MMAE payloads were mixed with the reduced mAb with payload:mAb molar ratio of 6.6 and 4.4, respectively, and incubated at 4°C for 1 h. The reaction was terminated by adding 20-fold molar excess of cysteine over payload and the final products were purified by G-25 gel filtration. In *in situ* conjugation, 7 equivalent of tris (2-carboxyethyl) phosphine (TCEP) was used to reduce 5 mg/mL mAb in 50 mM borate buffer, pH 8.0. The payload was added simultaneously with TCEP at 7 equivalent. After incubation at 37°C for 2 h, the product was purified by G-25 gel filtration.

### Characterization of ADCs

#### Drug-antibody ratio and structure

The integrity of ADC structure was analyzed using SDS-PAGE. The average drug-antibody ratio (DAR) was calculated using the following equation [21, 22]:

$$\text{DAR}=\frac{\varepsilon_{\text{mAb}}^{248\;\text{or}\;252}-{R\varepsilon }_{\text{mAb}}^{\text{280}}}{{R\varepsilon }_{\text{Drug}}^{\text{280}}-\varepsilon_{\text{Drug}}^{248\;\text{or}\;252}}$$ (1)

Where R = A_248_/A_280_ = Absorbance ratio. ε_mAb_^248/252^ = 9.41×10^4^ M^-1^cm^-1^, ε_mAb_^280^ = 2.34×10^5^ M^-1^cm^-1^, ε_MMAE_^248^ = 1.5×10^3^ M^-1^cm^-1^, ε_MMAE_^280^ = 1.59×10^4^ M^-1^cm^-1^, ε_DM1_^252^ = 2.64×10^5^ M^-1^cm^-1^, ε_DM1_^280^ = 5.23×10^3^ M^-1^cm^-1^. The UV absorbance was measured using a NanoDrop 2000 Spectrophotometer.

#### Surface binding and internalization

The live-cell confocal laser scanning microscopy technique was utilized to evaluate the surface binding capability and internalization of mAb and ADC in HER2^+^ BT474 cell line. The BT474 cells were seeded on glass coverslips (Warner Instruments, Hamden, CT) and transduced with BacMam 2.0 CellLight Late Endosomes-RFP and BacMam GFP Transduction Control to stain late endosomes and cytoplasm of BT474 cells, respectively, overnight. The transduced cells were rinsed twice with PBS buffer, stained with 2 μg/mL Alexa Fluor 647 labeled anti-HER2 mAb in a PBS buffer containing 10% inactivated goat serum and 1% bovine serum albumin (BSA), and incubated at 37°C under the microscope. The dynamic imaging profiles were captured using a confocal microscope (Olympus IX81, Center Valley, PA) every 20 min until ADC trafficked to late endosomes for lysosomal degradation to release drugs intracellularly.

#### Anti-breast cancer toxicity evaluation

The HER2^+^ BT474 cells and control cells were seeded in 96-well plates with seeding density of 0.05x10^6^ cells/mL in 75 μL DMEM/F12 or DMEM complete medium, and incubated at 37 ^o^C for 24 h. Equal volume of medium containing ADCs, free drugs (positive control), or PBS (control) was added to the well-plate cultures to initiate the anti-cancer toxicity study. After incubation at 37 ^o^C for 3 days, the culture volume in well plate was measured. The working solution of CellTiter-Glo Luminescent Cell Viability Assay Kit (Promega, Madison, WI) was added at equal amount before reading the luminescence with a Synergy H1 Hybrid Multi-Mode Microplate Reader (BioTek, Winooski, VT). The luminescent signal was proportional to the number of cells, and used to calculate the relative viability in each treatment.

## Results

### mAb production and purification

**Fig 2:**
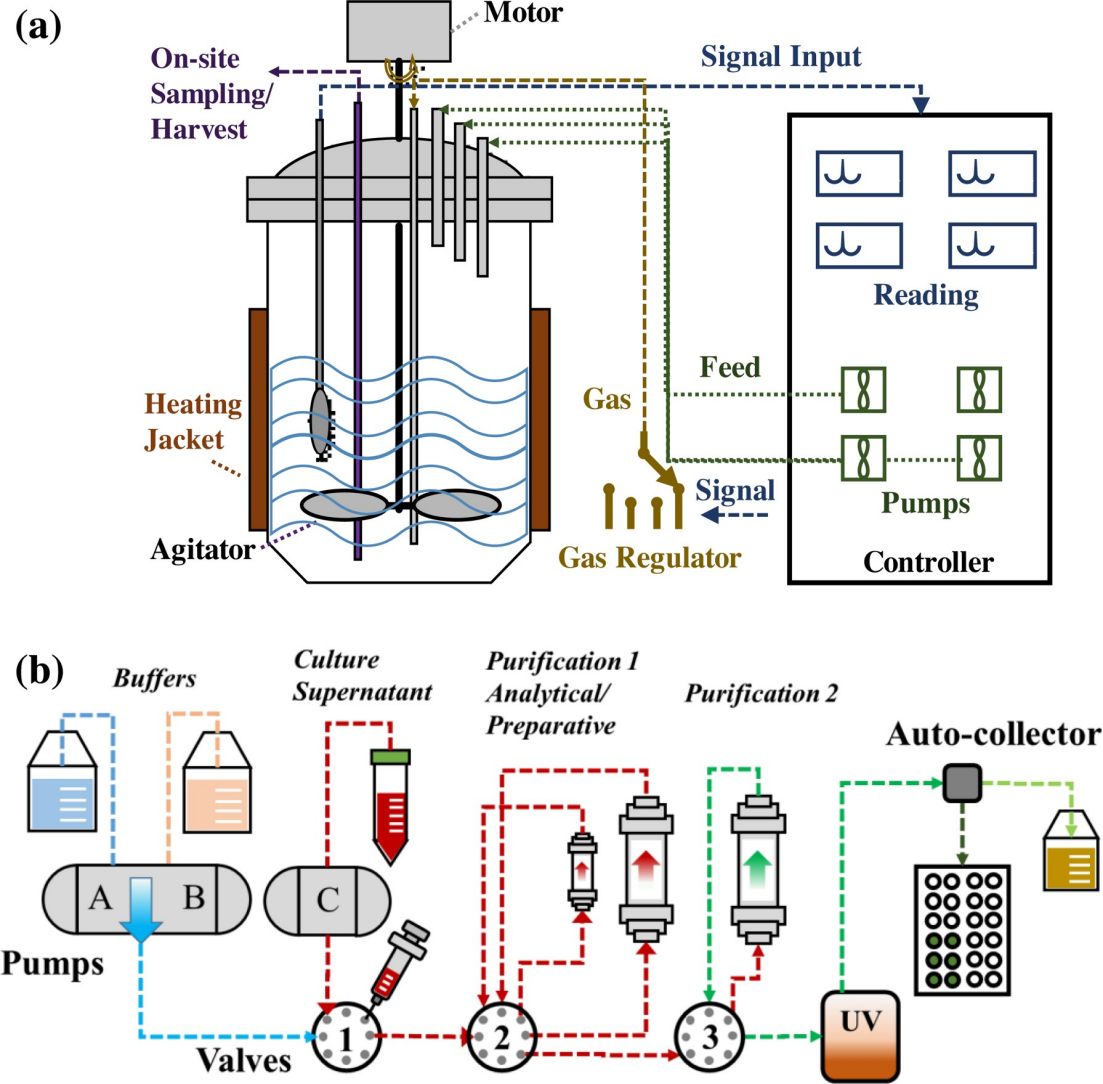
Anti-HER2 mAb production and purification. (**a**) Fed-batch production in stirred-tank bioreactor. (**b**) The purification process of mAb.

Feeding cell culture nutrients and accurately controlling bioproduction process parameters are important to improve mAb production. In this study, the anti-HER2 mAb was produced by CHO DG44/IgG from fed-batch cell culture in both shaker flask and stirred-tank bioreactor. Fig 2A showed the diagram of stirred-tank bioreactor connected to the automatic control panel of temperature, pH, DO and agitation, gas stations, and feeding pumps. Fig 2B showed the flowchart of mAb purification using NGC chromatography system.

#### Production

**Fig 3:**
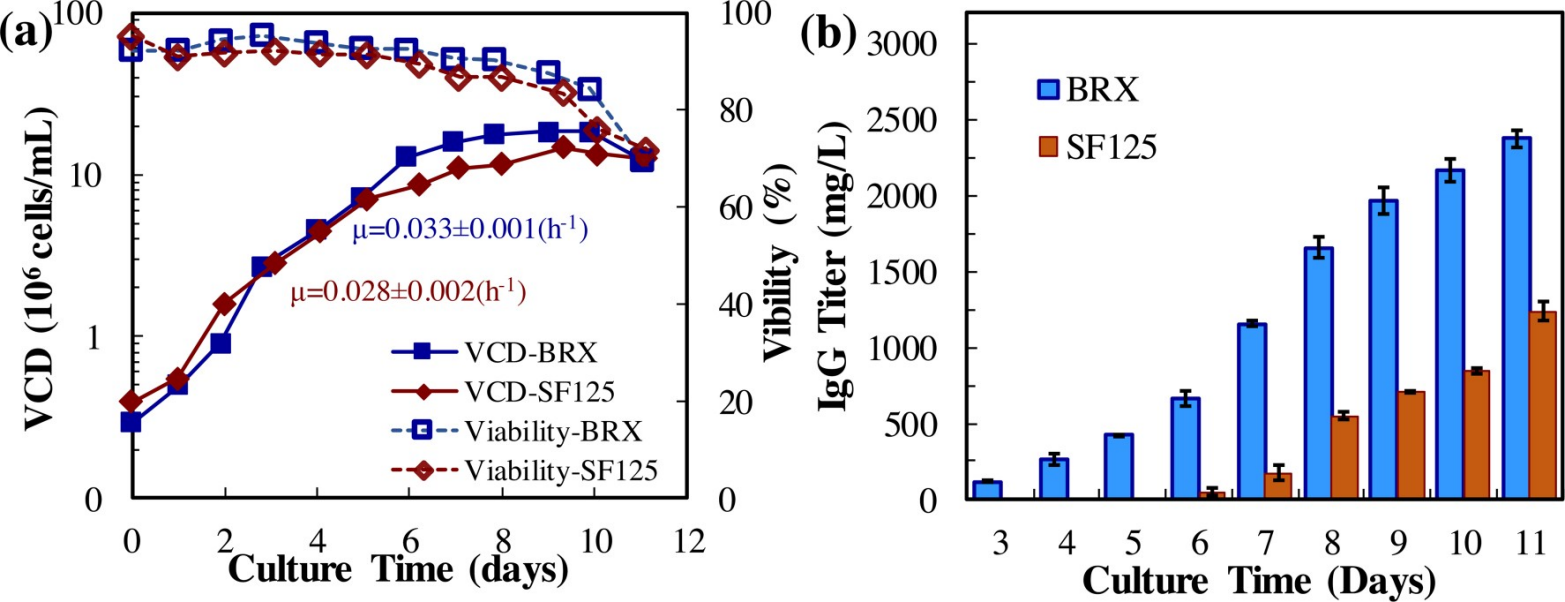
Anti-HER2 mAb production in fed-batch cell culture. (**a**) CHO/IgG (anti-HER2 mAb) cell growth in shaker flask (SF125) and 2-L stirred-tank bioreactor (BRX). Red rhombus: SF125 with working volume of 30 mL, Temp 37 ^o^C, agitation 130 rpm, and CO_2_ 5%. Blue square: BRX with working volume of 1 liter, Temp 37 ^o^C, agitation 70 rpm, and DO 70%. (**b**) Anti-HER2 mAb production.

The kinetics profiles of CHO cell growth and mAb production were presented in Fig 3 and the production parameters were summarized Table 1. Both shaker flask culture and bioreactor culture effectively produced mAb within 11 days. It was found that the specific growth rate was μ = 0.028±0.002 h^-1^ in shaker flask and 0.033±0.001 h^-1^ in bioreactor. The VCD in bioreactor was 18.1x10^6^ cells/mL, which was slightly higher than the VCD of 14.3x10^6^ cells/mL in shaker flask. The final anti-HER2 mAb titer was 2335.2±56.3 mg/L and 1278.2±62.5 mg/L, and the specific production rate was 30.00±2.14 pg/cell/day and 19.60±0.55 pg/cell/day in bioreactor and shaker flask, respectively. It is clear that the mAb production was improved by 58% and cell growth was increased by 26% in bioreactor as compared to shaker flask.

**Table 1:** Summary of fed-batch cell culture in bioreactor.

Cell culture	Shaker flask	Bioreactor
**μ (h**^**-1**^**)**	0.028±0.002	0.033±0.001
**VCD**_**Max**_ **(x10**^**6**^ **cells/mL)**	14.26±0.63	18.12±0.48
**mAb**_**Max**_ **(mg/L)**	1278.2±62.5	2335.2±56.3
**qmAb (pg/cell/day)**	19.60±0.55	30.00±2.14
**qGlucose (pg/cell/day)**	-137.65±2.19	-162.90±5.80

#### mAb characterization

**Fig 4:**
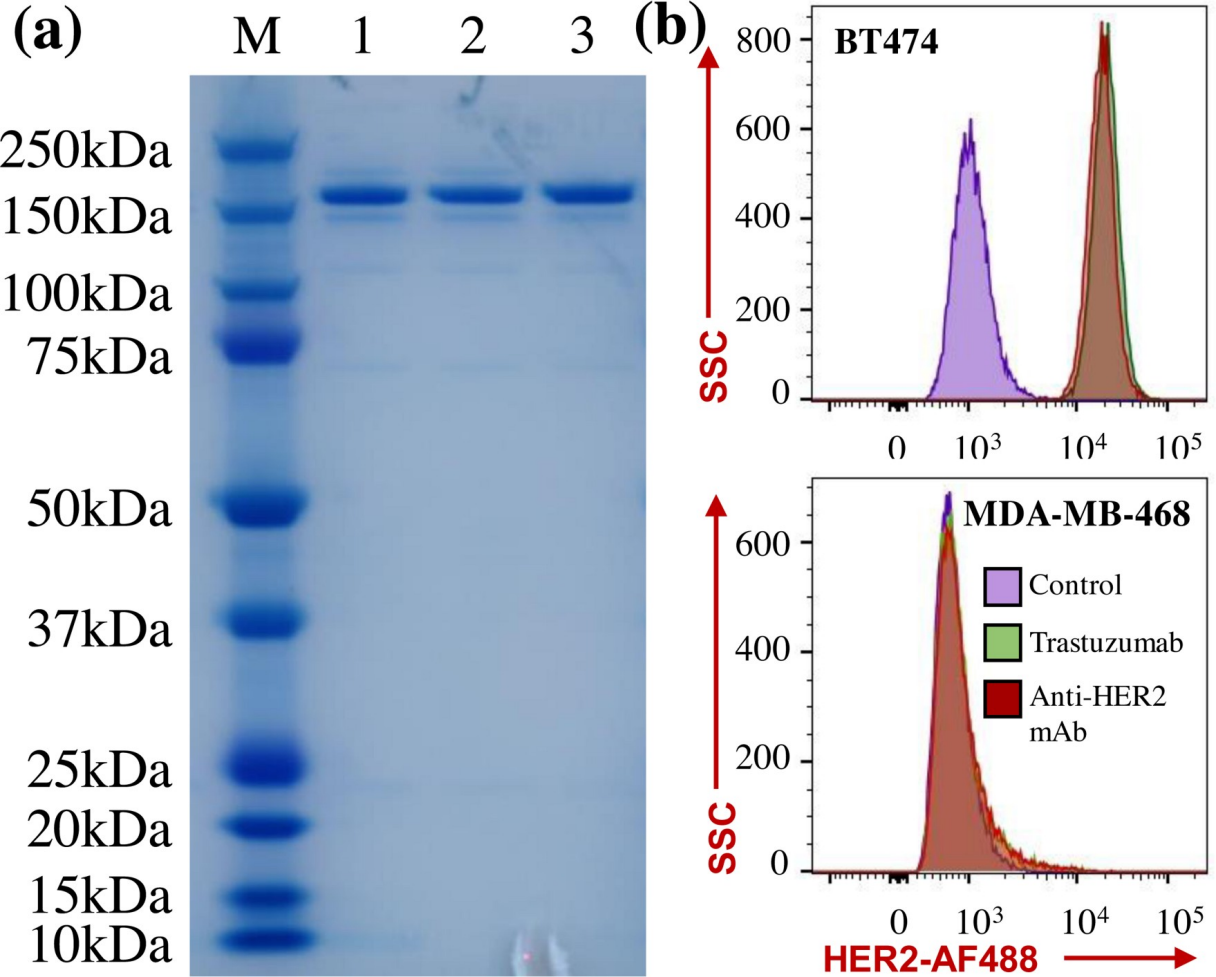
Evaluation of mAb purity and surface binding to HER2 receptor. (**a**) SDS-PAGE gel. M: marker, 1: anti-HER2 mAb purified by small-scale protein A purification kit, 2: anti-HER2 mAb purified by large-scale NGC system, 3: FDA approved Trastuzumab. Protein samples were loaded with 1 μg/well. (**b**) Flow cytometry analysis of receptor binding of purified anti-HER2 mAb and Trastuzumab to HER2^+^ BT474 cell line and negative control MDA-MB-468 cell line. Staining conditions: Alexa Fluor (AF) 488-labeled mAb was incubated with cells at 1 μg/million cells on ice for 30 min.

The purified anti-HER2 mAbs were characterized using SDS-PAGE gel together with the FDA approved Trastuzumab (Fig 4A). The results showed that our in-house anti-HER2 mAb had an expected protein size of 150 kDa and similar purity as Trastuzumab. A strong surface binding of mAb to the HER2 receptor is critical to achieve a high anti-cancer toxicity or efficacy of HER2-targeting ADC and to minimize the side effects caused by the non-specific targeting. Flow cytometry analysis was performed to quantitate and compare the cell surface binding of our anti-HER2 mAb and Trastuzumab to BT474 and control cells. Fig 4B revealed that Trasuzumab showed strong surface binding to BT474 cells but no binding to negative control cells, and our in-house anti-HER2 mAb had similar surface binding as Trasuzumab. These data indicated that the generated ADC had strong and specific surface binding to HER2 receptor in breast cancer.

### Bioprocess development of ADC construction

In this study, we evaluated the factors that affected ADC yield and quality, including potent drugs; conjugation approaches; molar ratio among drug, linker, and mAb; linker selection; and product purification.

#### Potent drugs

**Fig 5:**
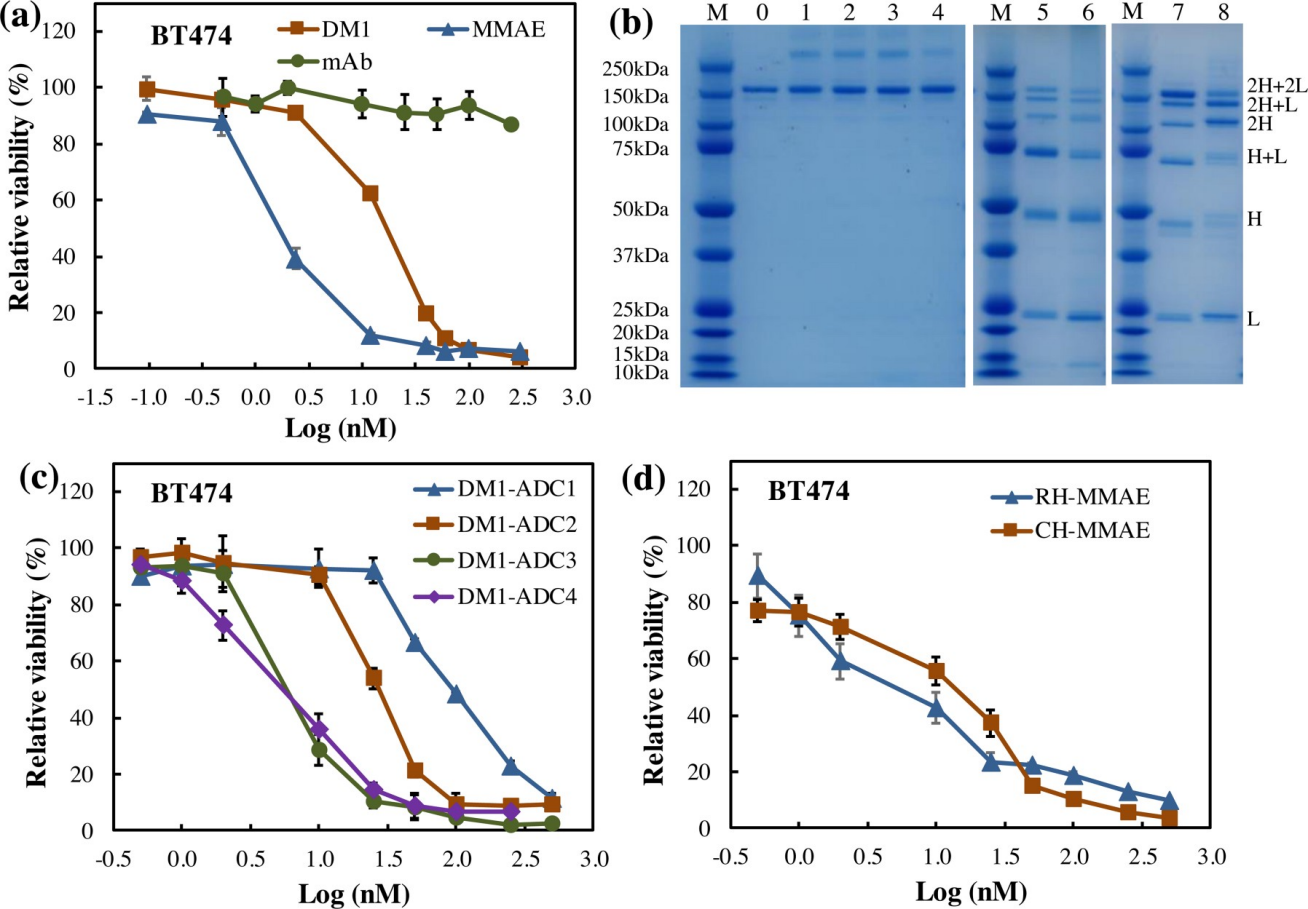
Evaluation of ADCs constructed in different production processes. (**a**) Anti-cancer toxicity of free drugs. Blue triangle: MMAE, Red square: DM1, Green circle: mAb. (**b**) SDS-PAGE of ADCs. M: marker, 0: purified mAb, 1–4: lysine-based DM1-carrying ADCs (named as DM1-ADC1-4) with drug:linker:mAb ratio of 4:4:1, 8:4:1, 8:4:1, and 16:8:1, respectively. DM1-ADC1 and ADC2 were purified using G25 column, and DM1-ADC3 and ADC4 were purified using protein A column. 5–8: Cysteine-based ADCs. 5: ADC from sequential conjugation with rebridging linker. 6: ADC from sequential conjugation with traditional linker. 7: ADC from *in situ* conjugation with rebridging linker (named as MMAE-ADC1). 8: ADC from *in situ* conjugation with traditional linker (named as MMAE-ADC2). ADC samples were loaded to SDS-PAGE gel with 2 μg/well. (**c**) Anti-cancer toxicity of lysine-based anti-HER2 mAb-DM1 ADCs. Blue triangle: DM1-ADC1, Red square: DM1-ADC2, Green circle: DM1-ADC3, Purple rhombus: DM1-ADC4. (**d**) Anti-cancer toxicity of anti-HER2 mAb-MMAE ADCs. Blue triangle: MMAE-ADC1, Red square: MMAE-ADC2.

Two potent chemical drugs, i.e. MMAE and DM1, that induce apoptosis by blocking the polymerization of tubulin, were used to investigate the cysteine- and lysine- based conjugation production process [5, 23]. Fig 5A described the dose-dependent anti-breast cancer toxicity using free drugs. It is shown that the viability of HER2^+^ BT474 cells was reduced by MMAE to 12% at concentration of 12 nM and 6% at 60 nM, and the viability was decreased by DM1 to 62% at 12 nM and 11% at 60 nM. As a negative control, mAb was not toxic to breast cancer cells in this study.

#### Conjugation approach

In lysine-based conjugation, the Sulfo-SMCC linker reacted with the 10 chemically accessible lysine residues in mAb, and generated mAb-DM1 ADCs with DARs of 0–10. As shown in the SDS-PAGE gel (Fig 5B), the structure of mAb in ADC was not obviously changed by conjugation at lysine. In cysteine-based conjugation, the cysteine was reduced to generate free thiol groups and generated ADCs with DARs of 2, 4, 6 or 8. Although the attractive non-covalent bonds could maintain the structure of mAb [24], the break of inter-chain disulfides significantly reduced the stability of ADCs, which was confirmed by the heterologous structure of mAb in ADCs (Fig 5B).

#### Molar ratio of drug:linker:mAb

Three different ratios of drug:linker:mAb (4:4:1, 8:4:1 and 16:8:1) were evaluated in the lysine-based conjugation and generated three DM1-carrying ADCs, including DM1-ADC1, DM1-ADC2 and DM1-ADC4. The DARs of these three ADCs were 3.15±0.20, 3.68±0.10 and 4.51±0.13, respectively. It is clear that DAR was increased by 16% when the drug amount doubled, and increased by 36% when the drug amount quadrupled and linker amount doubled. These DAR data were consistent with previous studies [15, 21]. Fig 5B revealed that all these ADCs had integral structure although a small portion of aggregation was observed, which could be caused by the hydrophobicity of the linker and payload [15]. The ADC4 showed a higher anti-breast cancer toxicity with IC_50_ value of 3.88 nM than that of ADC1 with IC_50_ value of 63.16 nM and ADC2 with IC_50_ value of 23.67 nM (Fig 5C). Therefore, the higher ratio of drug and linker in the lysine-based conjugation improved the DAR and anti-cancer toxicity of ADC.

#### Purification method

In addition to G25 column, protein A column was also tested in ADC purification. After lysine-based conjugation using the same drug:linker:mAb of 8:4:1, DM1-ADC2 and DM1-ADC3 were purified using G25 and protein A, respectively. The recovery rate of DM1-ADC2 was 96.1±4.8%, much higher than the recovery rate (65.8±5.9%) of DM1-ADC3. However, DM1-ADC3 showed higher cytotoxicity than DM1-ADC2, with IC_50_ of 5.07 nM vs. IC_50_ of 23.67 nM (Fig 5C). These results indicated that G25 column significantly improved ADC recovery rate but slightly reduced the anti-cancer toxicity as compared to protein A column.

#### Rebridging linker

A rebridging linker that can cross link the reduced cysteine was employed to maintain the mAb structure in cysteine-based ADC (Fig 1C). It is found that the rebridged ADC had less single chain, i.e. 2H, H and L (Fig 5B), and also showed higher cytotoxicity than the non-bridged ADC (Fig 5D).

#### Sequential vs in situ conjugation

Both sequential and *in situ* conjugations were applied in the construction of mAb-MMAE using rebridging linker and traditional linker. The SDS-PAGE showed that the *in situ* conjugation significantly increased the production of ADC via improving the content of stable structure (2H+2L, 2H+L, 1H+L, H+L).

### Binding and internalization of ADC

**Fig 6:**
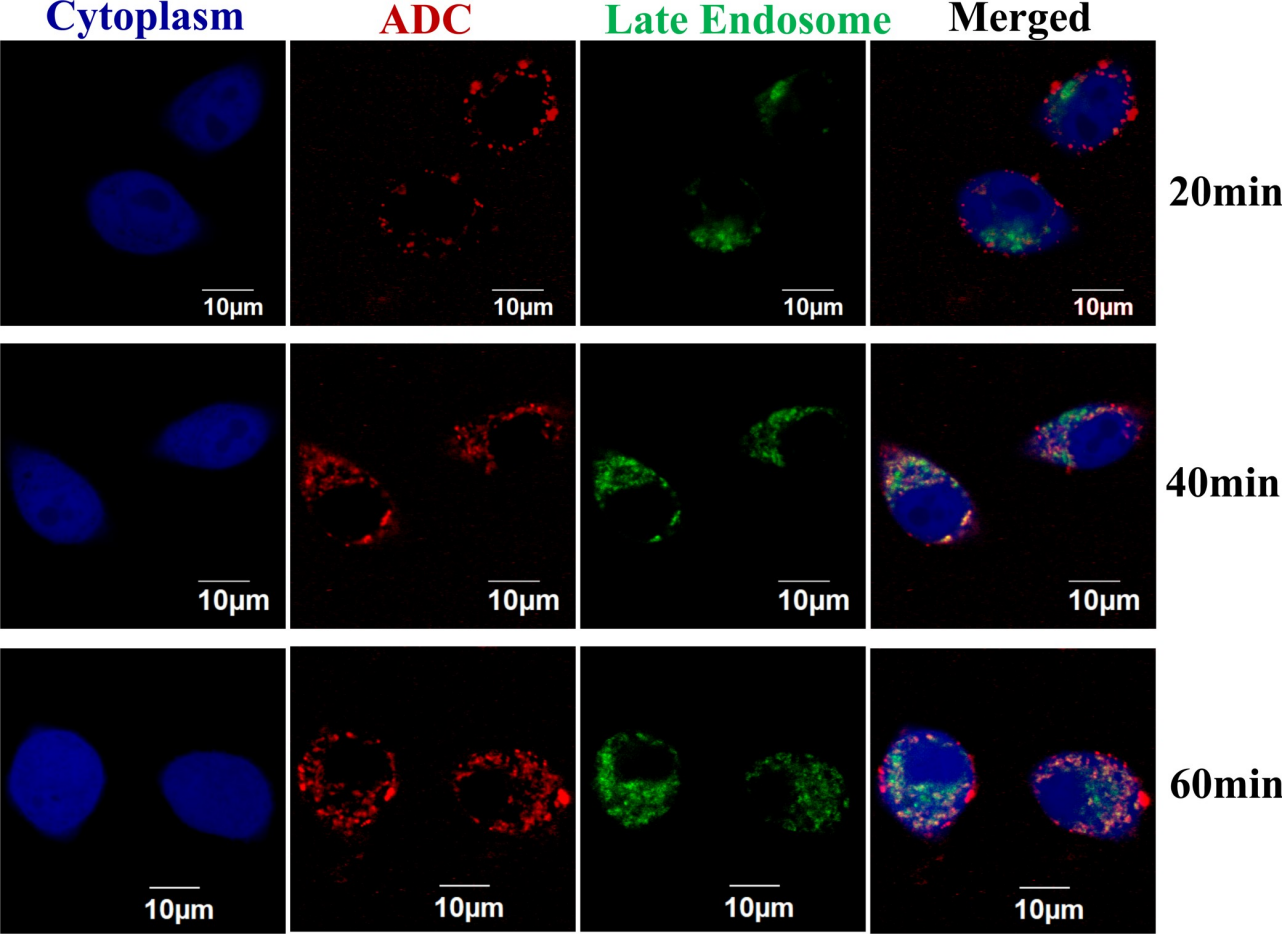
Surface binding and internalization process of ADC by confocal laser scanning microscopy. The BT474 cells were transduced with BacMam 2.0 CellLight Late Endosomes-RFP and BacMam GFP Transduction Control to stain late endosomes (green) and cytoplasm (blue), respectively. The DM1-ADC4 (red) was labeled with Alexa Fluor 647 and stained cells at 2 μg/mL in PBS buffer containing 10% inactivated goat serum and 1% BSA. The cytoplasm, late endosome, and ADC were excited by lasers with wavelength of 488 nm, 543 nm, and 633 nm.

Live-cell confocal microscopy imaging technique was used to monitor the surface binding and internalization of ADC in breast cancer cell. The dynamic profiles of confocal imaging were presented in Fig 6. No binding or internalization was observed on the negative control MDA-MB-468 cells, which is consistent with previous study [25]. After mixing the AF647 labeled anti-HER2 mAb-MMAE ADC (red color) with the HER2^+^ BT474 cells (blue color), the ADC bound to cell surface within 20 min. Then the ADC is quickly internalized, which is triggered by the receptor-mediated endocytosis [26], properly localized at late endosome (green color) for lysozyme degradation within 40 min [27], and continuously accumulated intracellularly within 60 min [28].

## Discussion

The production process of ADC can significantly affect its quality and yield [29]. This study used HER2-targeting ADC as a model therapeutics to evaluate and optimize the ADC bioproduction process, including mAb production, linker selection, conjugation approach, and purification method.

The mAb enables ADC to specifically target the surface receptor in cancer cells. The high quality of mAb, such as glycosylation, sialylation and stability, can improve the biological function of ADC, and the high productivity can significantly reduce the development and production cost of ADC. In this study, a high-titer and high-quality HER2-targeting mAb was produced from a fed-batch cell culture in stirred-tank bioreactor. Fed-batch process has been widely used in mAb production, which can regulate its post-translational modification and productivity [30, 31]. The flow cytometry analysis and confocal microscopy imaging demonstrated that our mAb had strong and specific surface binding capability.

Previous studies showed that the heterogeneity of DAR could diminish the *in vivo* solubility, impair binding capability, and influence pharmacokinetic/pharmacodynamic efficacy of ADC [21, 32–34]. This study showed that our lysine-based ADC conjugation process generated ADCs with a good range of DAR (i.e. 3.1–4.5). The cysteine-based conjugation caused the structural loss of disulfide bonds, which caused a high heterogeneity of ADC. Several strategies were developed to impair the ADC structure caused by cysteine reduction, such as engineering cysteine residue [35, 36], introducing unnatural amino acids [37], and utilizing additional enzymes in ADC conjugation process [38, 39]. However, these techniques were time consuming and had limited application scenarios. The linker bridging technique was developed to repair ADC structure, optimize DAR and simplify conjugation operation [17, 20, 40]. This study used the rebridging linker in cysteine-based conjugation, which improved the ADC integrity and anti-cancer toxicity. Literature also reported that the ADC constructed with non-cleavable linker showed higher anti-cancer toxicity and stability *in vitro* [41], and improved anti-cancer efficacy and pharmacokinetic performance *in vivo* [25, 42]. Therefore, novel linker development is an effective approach to optimize the bioproduction process of ADC. The improved integrity and stability of ADC indicates higher anti-tumor efficacy and better pharmacokinetics [17, 35], which will be further evaluated *in vivo* using xenografts mouse model in future.

The comparison between our study and reported data was summarized in Table 2. It is clear that the ADCs that were prepared with the optimized process showed high anti-cancer toxicity and the IC_50_ values were similar to previous publications, but the viability of cancer cells post treatment was lower than most reported data, indicating a better cytotoxicity. In addition, the anti-cancer toxicity was affected by ADC preparation process, targeted cell line, treatment timeline, detection assay, etc.

**Table 2:** Summary of ADC cytotoxicity assay.

Toxicity	DM1-ADC		MMAE-ADC	
	This Study^a^*	Literature*	This study^a^*	Literature
**IC**_**50**_ **(nM)**	3.88	2.71–4.26^a^ and 0.24^b^ [25], 4.7^a^ [43]	1.95	2.7–13.8^c^** [44], 0.60^a^** [45]
**Viability (%)**	<10	50–60^a^ and 30–35^b^ [25], 40^b^ [46]	<10	5–15^c^** [19], 25^a^** [45]

**Note:** Cell lines:

^a^BT474,

^b^SK-BR-3, and

^c^ Karpas 299;

assay timeline:

*3 days and

**4 days.

## Conclusions

ADC is a promising targeted therapy for cancer treatment. This study developed a robust ADC production process by investigating mAb production and conjugation conditions. The collected results or observations can be used to guide ADC development and production, which will accelerate the ADC-based anti-cancer therapy development and eventually benefit cancer patients.
